# Characterization of DBP contamination in a soil-wheat system and variety-specific tolerance associated with rhizosphere microbial communities

**DOI:** 10.1186/s12870-026-08762-w

**Published:** 2026-04-22

**Authors:** Bingye Yang, Hongfei Zhang, Zhexuan Tan, Taibo Liang, Yanling Zhang, Huaxin Dai

**Affiliations:** 1 Zhengzhou Tobacco Research Institute of CNTC, Zhengzhou, 450001 PR China; 2 China National Tobacco Quality Supervision and Test Center, Zhengzhou, 450001 China

**Keywords:** Dibutyl phthalate (DBP), Contamination characteristics, Wheat varieties, Rhizosphere microorganisms

## Abstract

Phthalate esters (PAEs) are a class of widely prevalent environmental pollutants that pose a serious threat to farmland ecosystems and agricultural product safety. The results of this study showed that dibutyl phthalate (DBP) and di(2-ethylhexyl) phthalate (DEHP) were the main PAEs in wheat field soils. All six PAEs were detected in various wheat tissues, and DBP exhibited an accumulation pattern during its migration toward the grains. Therefore, using DBP as the stress factor, we successfully screened a strongly tolerant variety RC69 and a sensitive variety ZM7698 from 51 wheat varieties. Greenhouse experiments confirmed that the aboveground biomass of RC69 remained stable under DBP stress, while that of ZM7698 showed concentration-dependent inhibition. Rhizosphere bacterial community structure of RC69 remained stable under DBP stress, but significant shifts in ZM7698, which was manifested in the decrease in the abundance of key taxa such as Proteobacteria and Pseudomonas, and the enrichment of DBP-tolerant taxa such as Gemmatimonadetes. Functional prediction analysis indicated that DBP stress significantly altered the potential ecological functions of rhizosphere bacteria in ZM7698, especially those associated with carbon and nitrogen cycling. This study provides a theoretical basis and a potential technical approach for harnessing rhizosphere microbial regulation to enhance crop stress resistance, offering a promising strategy for the safe utilization of contaminated soils and the clean production of agricultural products.

## Introduction

Phthalate esters (PAEs) are a class of plasticizers that enhance the plasticity, strength, and durability of materials [1]. Di-n-butyl phthalate (DBP), one of the most common plasticizers in the PAEs family, has been listed as a priority control pollutant by both the United States Environmental Protection Agency (USEPA) and the China National Environmental Monitoring Center [2, 3]. Due to its weak interactions (e.g., hydrogen bonding) with plastic polymers, DBP easily migrates from plastic products (such as agricultural films, packaging materials, and cosmetics) to the environment [4]. Studies have shown that DBP can be widely distributed in soil, water, and sediments through pathways such as atmospheric deposition, surface runoff, and sewage discharge [5]. Notably, in the Huang-Huai-Hai Plain (including Henan Province)—the largest wheat-producing area in China—the average concentration of DBP in farmland soils has reached 287 µg/kg [6]. This value significantly exceeds the soil safety threshold (81 µg/kg) established by the USEPA [7].

The ecotoxicity of DBP has been widely confirmed, demonstrating significant harmful effects on both plants and soil microorganisms. In terms of plants, DBP can inhibit seed germination [8], destroy root ultrastructure [9], interfere with photosynthesis [10], and reduce crop quality [11]. In terms of soil microorganisms, short-term exposure can change the β diversity of community structure [12], while long-term pollution may cause irreversible damage to soil microbial communities, thereby destroying the functional stability of soil ecosystems [13]. These toxic effects not only directly affect the growth, development, yield, and quality of crops, but may also have a more far-reaching impact on agricultural systems by changing the soil microecological balance.

Wheat (*Triticum aestivum* L.), as a major cereal crop, holds a prominent position and serves as a staple food source for more than half of the global population [14]. DBP can be transported from soil or water to wheat grains through the vascular system [15, 16]. A study has reported that the DBP content in wheat grains can reach 1.36 mg/kg in agricultural fields with long-term use of plastic mulch [17]. It is worrying that DBP may enter the human body through the food chain after accumulating in wheat grains, causing multiple health hazards. Previous studies have confirmed that DBP exposure is associated with endocrine disruption [18], thyroid dysfunction [19], male reproductive impairment [20], fetal growth retardation [21], and pulmonary histological lesions [22], posing a severe challenge to public health.

Research has found that there are interspecies differences in plant resistance to DBP. For example, a study by Ma et al. [23] on seven plant species—wheat, alfalfa, ryegrass, radish, cucumber, oat, and onion—revealed that alfalfa, ryegrass, and onion exhibited significantly higher tolerance to DBP than the other four species. However, the differences in DBP resistance between different wheat varieties have not been reported. Rhizosphere microbial communities have been regarded as the “second genome” of plants. These microbes can promote nutrient acquisition, synthesize stress-alleviating metabolites, and activate host defense pathways, and thus their structural and functional stability plays a key role in regulating plant stress resistance [24]. For example, upon sensing organic pollutants, plant leaves generate long-distance reactive oxygen species (ROS) signals to actively recruit beneficial rhizosphere microbiota, thereby enhancing plant resistance [25]. DEHP stress can specifically enrich PAE-degrading microbial communities in the rhizosphere, driving the formation of complex bacterial interaction networks and thereby improving the degradation efficiency of phthalate esters in soil [26]. Among the various fates of soil PAEs, microbial degradation is the primary pathway [27]. Previous study has shown that the degradation rate of DBP is mainly affected by the number of soil microorganisms [28]. In addition, some studies have shown that rhizosphere microorganisms such as *Bacillus* [29], *Pseudomonas* [30], and *Gordonia* [31] can degrade DBP to help plants alleviate stress. Therefore, exploring DBP resistance among wheat varieties and its links to rhizosphere microbial communities is key to understanding plant adaptation to DBP pollution.

Previous studies have shown that DBP inhibits wheat growth and disrupts soil microbial communities [13, 32]. However, most of these studies focused on physiological responses of single varieties, overlooking the microbiological mechanisms underlying varietal differences in DBP resistance. Additionally, how microbial communities mediate wheat’s tolerance to DBP remains a mystery, which limits the practical application of microbial regulation to improve crop resistance. We hypothesize that different wheat varieties exhibit varying tolerance to DBP, and that the stability and functional integrity of the rhizosphere microbial community are key determinants of DBP tolerance in wheat. In this study, the farmland in central Henan Province was taken as the research object, aiming to: (1) Evaluate the characteristics of DBP pollution in the soil-wheat system and tolerance differences among varieties; (2) Analyze the response mechanisms of rhizosphere microbial communities to DBP stress in a tolerant cultivar (RC69) and a sensitive cultivar (ZM7698); (3) Identify key microbial taxa involved in DBP resistance. This study revealed the synergistic mechanism of wheat variety-microbial interaction against DBP stress for the first time, providing a theoretical basis for enhancing the resistance of sensitive varieties through rhizosphere microbial regulation, which is of great significance for ensuring food security.

## Materials and methods

### Sample collection

**Fig. 1:**
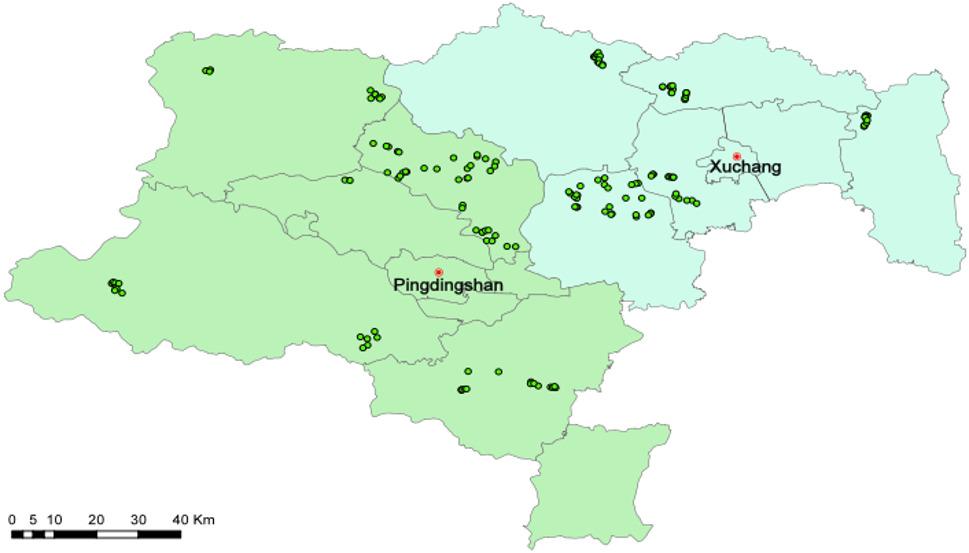
Spatial distribution of 156 soil sampling sites in central Henan Province, China. Note: This map was created using ArcGIS 10.8 based on the WGS84 coordinate system. The 156 soil sampling sites are represented by dark green dots, overlaid on administrative boundary basemap data. The red dot indicates the location of the city center. “Pingdingshan” and “Xuchang” are two cities in Henan Province, China

A total of 156 soil samples were collected from the main wheat-growing areas in Xuchang and Pingdingshan City, Henan Province, and the specific sampling sites were shown in Fig. 1. Sampling was conducted in late May 2020, corresponding to the maturity stage of wheat, and no fertilization, irrigation, or heavy rainfall events occurred within at least one week prior to sampling. According to the principles of random and multi-point mixing, GPS positioning and “S” sampling method were used to collect soil samples from 0 ~ 20 cm tillage layer, with each sample weighing approximately 1.5 kg. The collected soil samples were packed in cloth bags, brought back to the laboratory to be air-dried, and then packed in brown glass bottles after passing through a 100-mesh sieve for the determination of soil PAEs. When collecting soil samples from farmland, the corresponding wheat grain samples were also collected and placed in cloth bags, and brought back to the laboratory for PAEs content analysis. Throughout the experimental process, all plastic materials were avoided to prevent contamination.

### Determination of PAEs content

The concentrations of six PAEs (including DMP, DEP, DBP, BBP, DEHP, and DnOP) in soil and wheat grains were determined using gas chromatography-mass spectrometry (GC-MS). To avoid contamination, all plastic materials were excluded during the experiment, and glassware was treated with potassium dichromate cleaning solution. The samples were ultrasonically extracted with a dichloromethane: acetone (3:1, V/V) mixture, and then 50 µL of deuterated BIDP internal standard (10 mg/mL) was added to the sample. Standard solutions were prepared by diluting a 1 mg/mL mixed PAEs stock solution to concentrations of 100, 50, 25, 10, and 4 µg/mL, each spiked with 50 µL of the internal standard. GC-MS analysis was performed using a fused-silica capillary column (30 m × 0.25 mm × 0.25 μm) with a 5% phenyl/95% dimethyl polysiloxane stationary phase. The carrier gas was helium, and a splitless injection mode was applied with an injection volume of 1 µL. The inlet temperature was set at 250℃, the column flow rate at 0.80 mL/min, the detector temperature at 280℃, and the electron ionization ion source was used for detection.

### Screening of wheat varieties tolerant to DBP

The seed germination experiment was conducted following the method of Dai et al. [33] with modifications. A total of 51 wheat germplasm resources, kindly provided by the Henan Academy of Agricultural Sciences, were used in this study. The seeds were surface-sterilized with 2% H_2_O_2_ for 30 min, then rinsed three times with deionized water, and soaked for 2 h. The seeds were then placed in Petri dishes lined with two layers of moist filter paper, with 50 seeds per dish. Two treatments were set up: (1) Control group: 5 mL of pH 6.0 deionized water was added in per dish; (2) DBP treatment group: 5 mL of 50 g/L DBP was added. A DBP concentration of 50 g/L was selected to represent an acute, high-stress screening treatment rather than an environmentally relevant exposure. This concentration was determined based on preliminary experiments using a range of DBP concentrations (1, 5, 10, 25, 50, and 100 g/L), in which clear phenotypic differences were observed only at concentrations ≥ 50 g/L. All Petri dishes were incubated in a constant-temperature incubator at 25℃ for 48 h, after which the germination rate and root length were measured. Each treatment was performed with three biological replicates. The DBP tolerance of wheat varieties was evaluated using a weighted scoring method based on Wang et al. [34]. The relative root length (RRR) and the relative germination rate (RGR) were used as evaluation indicators, with weight coefficients of 0.5 each. The comprehensive tolerance score was calculated on a hundred-point scale. The comprehensive tolerance score for each wheat variety was calculated as follows:

$$\mathrm{Comprehensive}\;\mathrm{Score}\;=\left(\mathrm{RRR}\times0.5+\mathrm{RGR}\times0.5\right)\times100$$

where RRR = root length in DBP treatment / root length in control, and RGR = germination rate in DBP treatment / germination rate in control. All calculations were performed on a hundred-point scale, with higher scores indicating greater DBP tolerance.

### Greenhouse experimental design and methods

#### Experimental soil and DBP contamination treatment

Surface soil (0–20 cm) was collected from agricultural fields in Wangluo Town, Xiang County, Xuchang City, Henan Province. After removing stones and other impurities, the soil was thoroughly mixed, air-dried, and sieved through a 5 mm steel sieve. The physicochemical properties of the soil were as follows: pH 7.93, organic matter 12.12 g/kg, total nitrogen 0.87 g/kg, total nitrogen 0.87 g/kg, total potassium 14.25 g/kg, alkali-hydrolyzable nitrogen 76.54 mg/kg, available phosphorus 11.51 mg/kg, available potassium 125.20 mg/kg, Readily decomposable organic carbon 519.22 mg/kg, soil protein 2.53 mg/kg, and DBP content 0.21 mg/kg. The method for DBP contamination was referred to Gao et al. [35] with the following main steps: DBP was dissolved in acetone to prepare a 1000 mg/L standard solution, which was then sprayed onto 5 kg of soil and thoroughly mixed. The soil was placed in a cool, shaded area for 3 days to allow the acetone to evaporate. Subsequently, the soil collected in Wangluo Town was added in proportion to achieve nominal DBP concentrations of 0, 25, and 100 mg/kg (based on the amount of DBP added). It should be noted that the nominal concentrations represent the spiking levels. Following the 3-day equilibration period, the measured DBP concentrations in the prepared soils were 0.21 mg/kg (natural background level), 1.8 mg/kg (low-DBP group), and 13.1 mg/kg (high-DBP group).

#### Wheat cultivation and biomass measurement

Plump and uniformly sized wheat seeds were selected and sown in soils with different DBP concentrations: control (background soil), low DBP (1.8 mg/kg), and high DBP (13.1 mg/kg). Each treatment included three biological replicates, with each replicate consisting of one pot containing 10 plants. All pots were placed on the cultivation shelves in a completely randomized manner. Environmental conditions were strictly controlled throughout the experiment: the temperature was maintained at 22℃, the relative humidity was controlled at 60%, and the photoperiod was 14/10 hours (light/dark). All pots were irrigated with 300 mL of water every two days to maintain the consistency of soil moisture.

After 30 days of sowing, the aboveground parts of the plants were harvested, dried in a sterilizing oven at 55℃ for 48 h until constant weight was achieved, and then weighed to determine the aboveground biomass.

### High-throughput sequencing

Rhizosphere soil samples were collected following the method previously described by Yang et al. [36]. Total soil DNA was extracted from 18 rhizosphere samples (3 replicates per treatment) using the UltraClean Soil DNA Isolation Kit (Mobio Laboratories, Carlsbad, CA, USA) according to the manufacturer’s instructions. For Illumina MiSeq high-throughput paired-end sequencing, the bacterial 16S rDNA V4-V5 region was amplified using primers (515F: 5’-AATGATACGGCGACCACCGAGATCTACAC-NNNNNNNNTCTTTCCCTACACGACGCTCTTCCGATCT-GTGCCAGCMGCCGCGGTAA-3’) and barcode-specific primers with MiSeq adapters (926R: 5’-CAAGCAGAAGACGGCATACGAGAT-NNNNNNN-GTGACTGGAGTTC CTTGGCACCCGAGAATTCCA-CCGTCAATTCMTTTGAGTTT-3’). The fungal ITS region was amplified using specific primers (ITS1F: 5’-AATGATACGGCGACCACCGAGATCTAC AC-NNNNNNNN-TCTTTCCCTACACGACGCTCTTCCGATCT-CTTGGTCATTTAGAGGAAGTAA-3’) and barcode-specific primers with MiSeq adapters (ITS2R: 5’-CAAGCAGAAGACGGCATACGAGAT-NNNNNNNN-GTGACTGGAGTTCCTTGGCACCCGAGAATTCCA-GCTGCGTTCTTCATCGATGC-3’). The bacterial 16S rDNA Miseq high-throughput library and the fungal ITS Miseq high-throughput library were constructed by two-step PCR amplification. The first round of PCR amplification was conducted in a 50 µL system containing 25 µL of 2×Hieff Robust PCR Master Mix, 2 µL each of forward and reverse primers, and 50 ng of template DNA. Cycling conditions included initial denaturation at 94 °C for 3 min, followed by 27 cycles of 94 °C for 30 s, 50 °C for 1 min, and 72 °C for 30 s, with a final extension at 72 °C for 5 min. In the second round of amplification, Illumina bridge PCR compatible primers were used instead of primer R, and the reaction system was identical to that of the first round. High-throughput sequencing was performed on the Illumina MiSeq platform (Shanghai Majorbio Bio-pharm Technology Co., Ltd).

The raw sequencing data were demultiplexed using barcodes to assign reads to each sample. Trimmomatic software was used to remove low-quality sequences from the paired-end reads. Based on the overlap between paired-end reads, the FLASH software was employed to merge paired reads into a single sequence. At the same time, the Mothur software was used to control and filter the quality of the sequence, removing ambiguous bases, homopolymers, excessively long or short sequences, and chimeras generated during PCR amplification to obtain optimized sequences. In our study, a total of 972,159 raw sequences, 924,782 high-quality sequences, 261,574,775 base pairs and 1,058 Operational Taxonomic Units (OTUs) for fungi were obtained after screening and selecting. A total of 842,618 raw sequences, 725,171 high-quality sequences, 299,491,178 base pairs and 3,361 OTUs for bacteria were detected. OTUs were clustered at 97% similarity using the UPARSE software, and taxonomic annotation was performed by alignment against the SILVA 128 database. Based on the taxonomic information, statistical analysis of the microbial community structure was carried out at the taxonomic levels of phylum, class, order, family, genus and species.

### Data analysis

In the R environment (version 4.2.3), β diversity (based on Bray-Curtis dissimilarity PCoA) was calculated using the “phyloseq 1.36.0” package. Co-occurrence networks were constructed in R (v4.3.0) using the igraph package (v1.5.0) based on significant pairwise correlations (Spearman’s |ρ| > 0.6, FDR-adjusted *p* < 0.05) between microbial features. To focus on the most influential nodes while maintaining network clarity, the top 30 nodes ranked by degree were defined as highly connected nodes. The relative abundance data of differential microbes were standardized using Z-scores, and heatmaps were generated using the pheatmap package in R software (version 4.2.3). The ‘LEFSE’ command in Python 2 was used to conduct linear discriminant analysis (LDA), with significantly differential biomarkers displayed in a bar plot based on their LDA scores. The SIMPER analysis was performed using the simper() function in the vegan package within the R 4.2.3 environment. Functional annotation of bacterial relative OTU taxonomic table was performed with command collapse_table.py of FAPROTAX 1.2.10 [37]. Welch’s t-test was used to assess the significance of differences between the two treatment groups using STAMP software. Conventional bar charts, stacked plots, and scatter plots were generated using GraphPad Prism 8.

## Results

### Distribution characteristics of PAEs in agricultural soils

**Fig. 2:**
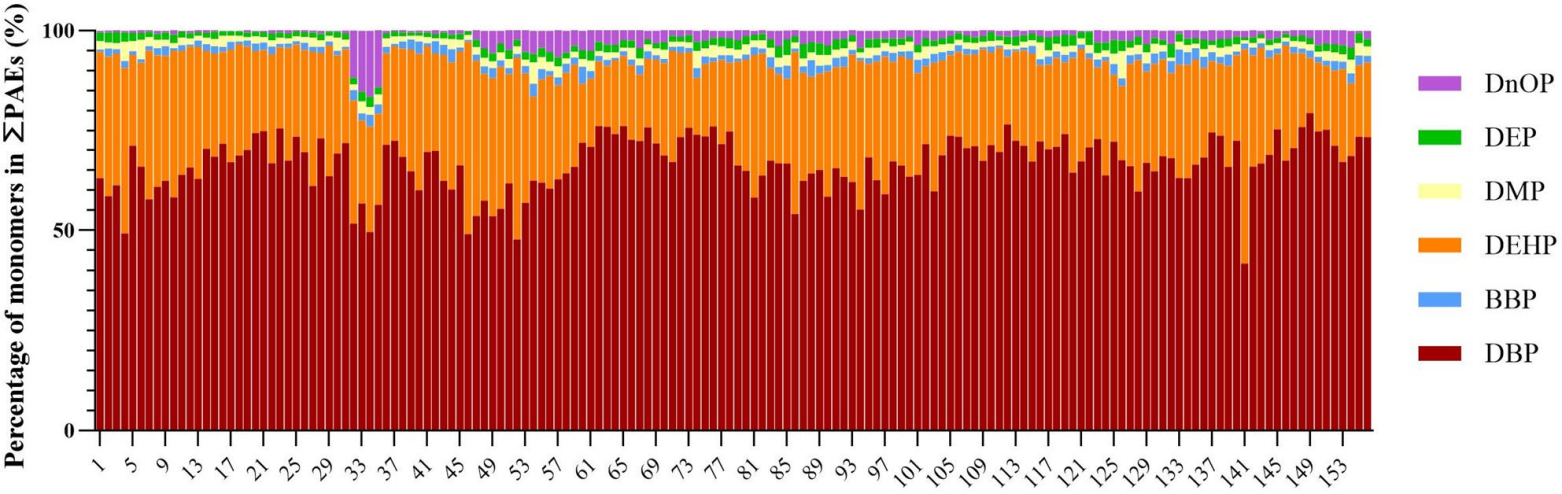
Composition proportions of six priority-controlled PAEs in soils. Note: Data show the percentage proportions of six priority-controlled phthalate esters (PAEs) (DBP, DEHP, DnOP, BBP, DMP, and DEP) relative to total PAEs in surface soils (0–20 cm) collected from 156 representative wheat fields in central Henan Province

In this study, six PAEs prioritized by the USEPA were measured in 156 wheat fields in central Henan Province. The results showed that DBP and di(2-ethylhexyl) phthalate (DEHP) were the main pollutants, accounting for over 75% of the total PAEs (Fig. 2). Specifically, DBP constituted more than 50% of the PAEs in 96.79% of the sampling sites, while DEHP exceeded 20% in 79.48% of the sites (Fig. 2). Notably, DBP even contributed to more than 70% of the total PAEs in 36.54% of the sampling sites.

### Accumulation of PAEs in the aboveground tissues of wheat

**Fig. 3:**
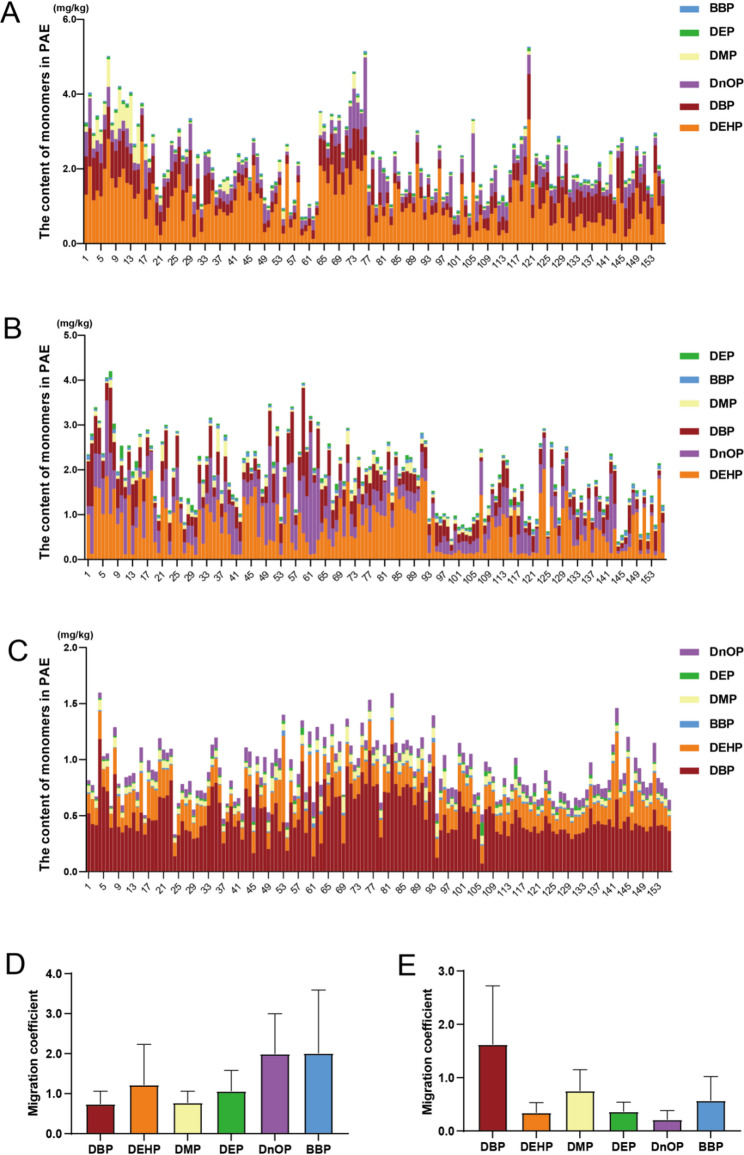
Concentrations of priority-controlled PAEs in the aboveground tissues of wheat. Note: (**A**) Concentrations of six priority-controlled PAEs in wheat leaves. **B** Concentrations of six priority-controlled PAEs in wheat stems. **C** Concentrations of six priority-controlled PAEs in wheat grains. **D** Migration coefficients of six priority-controlled PAEs from stems to leaves of wheat. **E** Migration coefficients of six priority-controlled PAEs from leaves to grains of wheat. Bar heights represent mean values, with each sample analyzed in triplicate

The detection results showed that PAEs contaminants were detected in all samples of wheat aboveground tissues collected from 156 sampling sites. In wheat stems, the total accumulation of six PAEs ranged from 0.811 to 5.471 mg/kg, with a mean value of 2.235 mg/kg (Fig. 3A). In wheat leaves, the total accumulation ranged from 0.4026 to 4.201 mg/kg, with a mean value of 1.867 mg/kg (Fig. 3B). In wheat grains, the total accumulation was the lowest, ranging from 0.334 to 1.598 mg/kg, with a mean value of 0.889 mg/kg (Fig. 3C). DBP and DEHP exhibited significant bioaccumulation dominance in grains, with average contents of 0.510 mg/kg and 0.209 mg/kg, accounting for 57.4% and 23.5% of the total PAEs, respectively (Fig. 3C). In the stem-to-leaf migration system, the migration coefficients of DEHP, DEP, BBP and DnOP were greater than 1, while those of DBP and DMP were less than 1 (Fig. 3D). In the leaf-to-grain system, only DBP had a migration coefficient greater than 1, and the other five PAEs were less than 1, indicating the enrichment of DBP in grains (Fig. 3E).

### Screening of DBP-tolerant wheat varieties

**Fig. 4:**
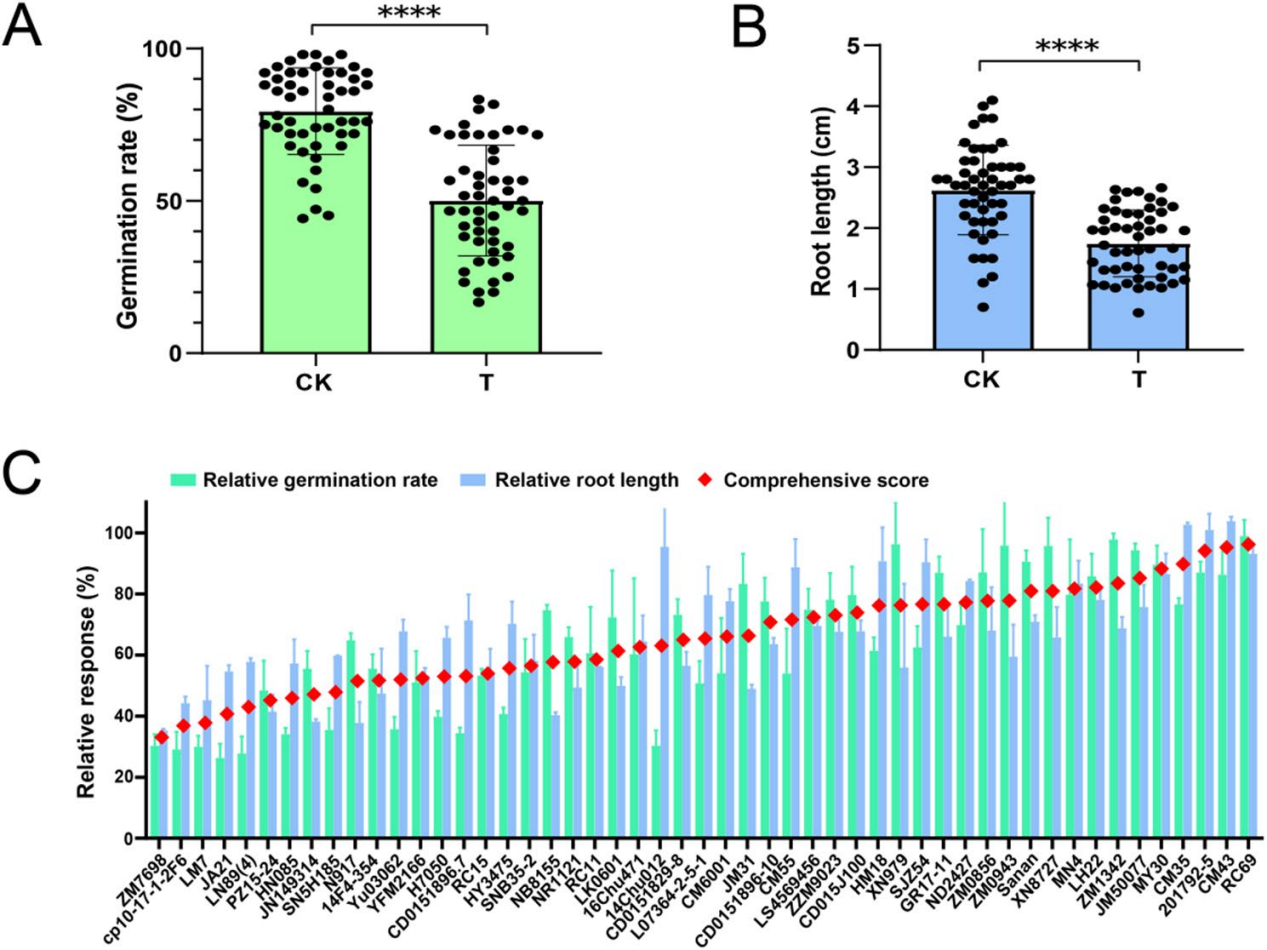
Evaluation of DBP tolerance based on germination and root length. Note: (**A**) Germination rate under control and DBP-treated conditions. **B** Root length under control and DBP-treated conditions. **C** Relative germination rate, relative root length, and comprehensive tolerance score used for DBP-tolerant variety screening. CK, Blank control; T, Treatment with 50 g/L DBP. Statistical significance was assessed using Student’s t test. **** indicates *P* < 0.0001

Based on the analysis of PAEs contamination characteristics in the soil-crop system (Figs. 2 and 3), DBP was selected as the target stress factor to evaluate the tolerance differences of 51 wheat varieties. The experimental results showed that DBP stress significantly inhibited wheat germination and root growth. Specifically, under the control conditions, the germination rates of 51 wheat varieties ranged from 44.24% to 98.00%, with an average germination rate of 79.45%. After exposure to DBP stress, the average germination rate decreased to 50.13%, representing a relative reduction of 36.9% (Fig. 4A). Similarly, under the control conditions, the root length of 51 wheat varieties ranged from 0.70 cm to 4.14 cm, with an average root length of 2.62 cm. DBP treatment decreased the average root length to 1.75 cm, a reduction of 33.2% (Fig. 4B).

The weighted evaluation results based on relative germination rate and relative root length showed significant differences in the comprehensive scores of DBP tolerance among 51 wheat varieties. Among them, variety RC69 showed the strongest DBP tolerance (comprehensive score: 96.07), which was higher than that of other varieties. In contrast, variety ZM7698 showed the weakest tolerance (comprehensive score: 32.83), displaying an extremely significant difference compared to RC69 (*P* < 0.01) (Fig. 4C).

## Varietal differences in biomass and internal DBP accumulation under DBP stress

**Fig. 5:**
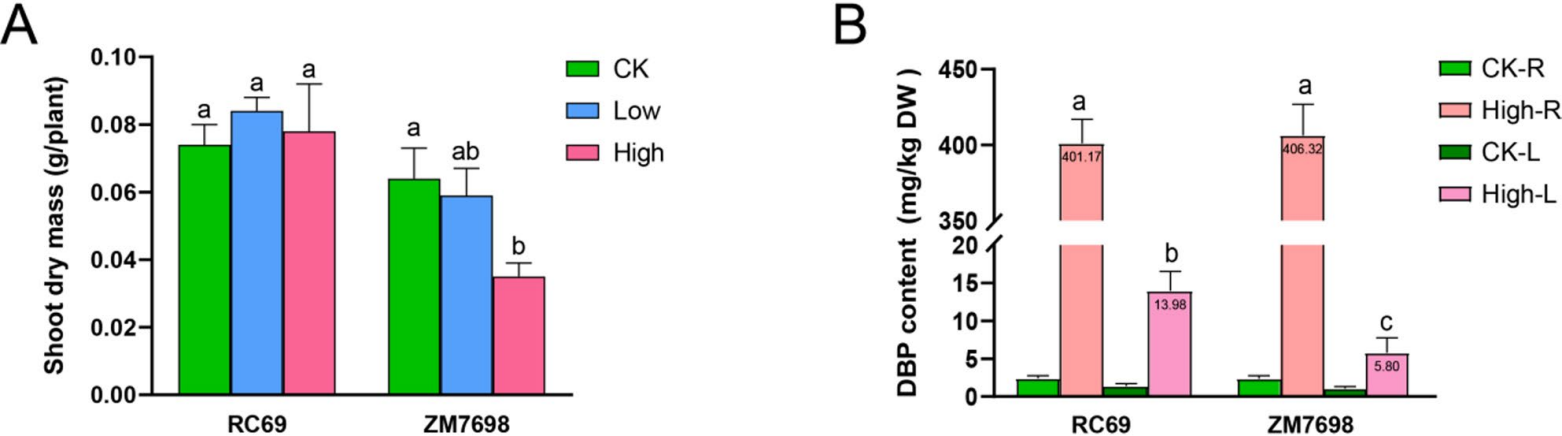
Plant responses to DBP stress. Note: (**A**) Shoot dry weight of tolerant (RC69) and sensitive (ZM7698) wheat varieties after 30 days of exposure to different DBP concentrations. **B** DBP accumulation in roots and leaves of RC69 and ZM7698 under high DBP stress. CK (control, background soil), Low (1.8 mg/kg DBP), High (13.1 mg/kg DBP), CK-R (roots, control), CK-L (leaves, control), High-R (roots, 13.1 mg/kg DBP), High-L (leaves, 13.1 mg/kg DBP). Data are presented as means + SE (*n* = 6). The different letters above the bars indicate significant difference, LSD test, *P* < 0.05

To validate the comprehensive evaluation results shown in Fig. 4, three DBP concentration gradients were set up in this study: control (background soil), low DBP (1.8 mg/kg), and high DBP (13.1 mg/kg). The tolerant variety RC69 and the sensitive variety ZM7698 were subjected to stress treatment under greenhouse conditions. The results showed that there was no significant difference in the shoot dry matter accumulation of variety RC69 among the treatments, and its biomass remained within the range of 0.074–0.084 g/plant, demonstrating stable stress resistance ability (Fig. 5A). In contrast, ZM7698 exhibited significant concentration-dependent inhibition. Compared with the control group, the biomass of the low DBP treatment group decreased by 8.20%, and that of the high DBP treatment group decreased by 45.31%, with statistically significant differences (Fig. 5A). Additionally, DBP accumulation in roots and leaves was measured under high DBP stress. While RC69 and ZM7698 showed comparable DBP levels in roots, the DBP content in RC69 leaves was significantly greater than that in ZM7698 (Fig. 5B). These results confirm that RC69 had a significant advantage in DBP tolerance, while ZM7698 displayed a concentration-dependent sensitive response to DBP stress.

### Analysis of microbial community structure in the rhizosphere soil of DBP-tolerant and DBP-sensitive wheat varieties

**Fig. 6:**
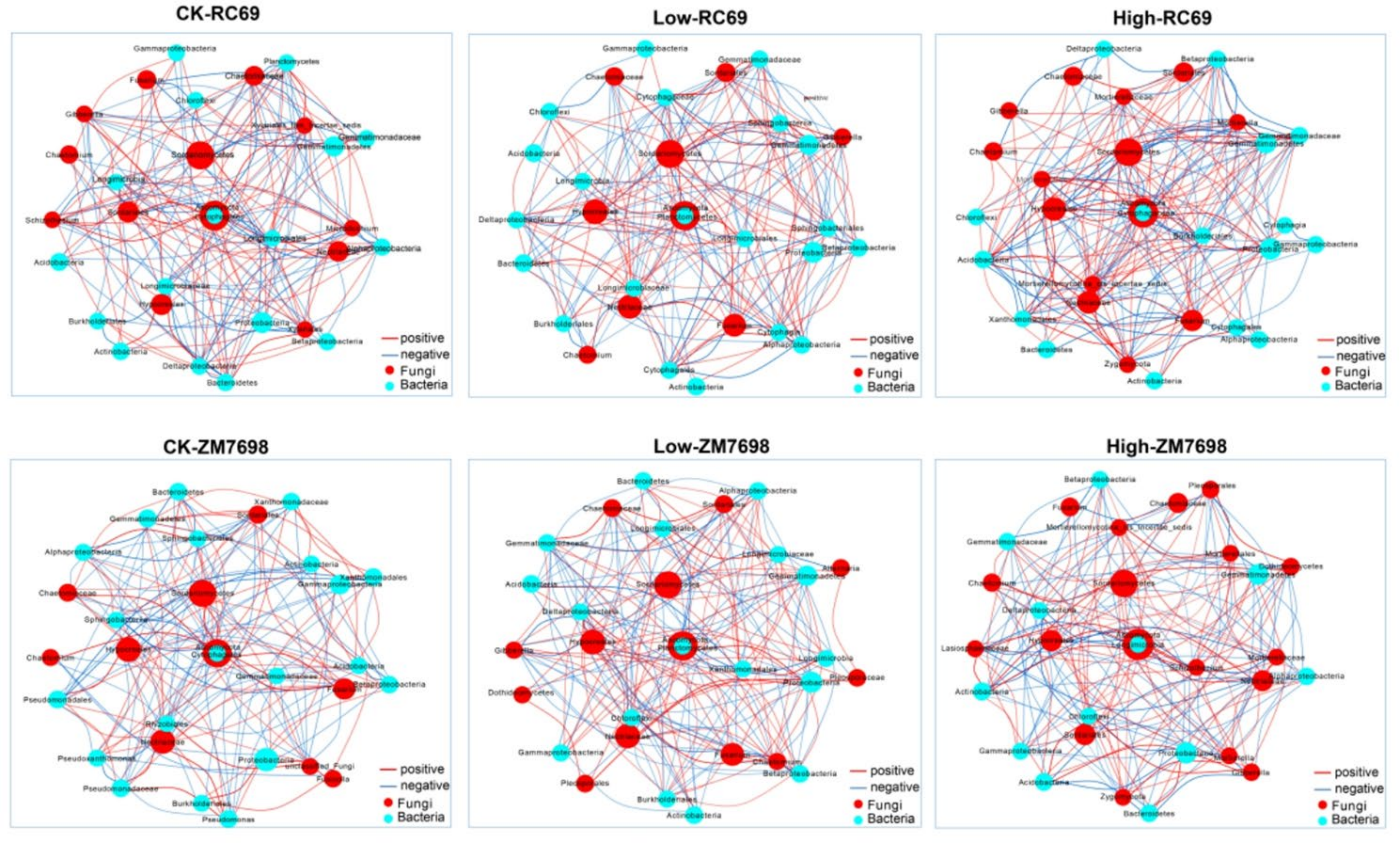
Soil bacterial co-occurrence network under DBP stress. Note: Nodes are colored by microbial type (blue: bacterial OTUs; red: fungal OTUs) and sized by the log10-transformed total abundance of each OTU across all samples. The network displays the top 30 OTUs with the highest total abundance. Edges represent significant correlations (Spearman |ρ| > 0.6, FDR-adjusted *p* < 0.05), with blue and red edges indicating positive and negative correlations, respectively

**Fig. 7:**
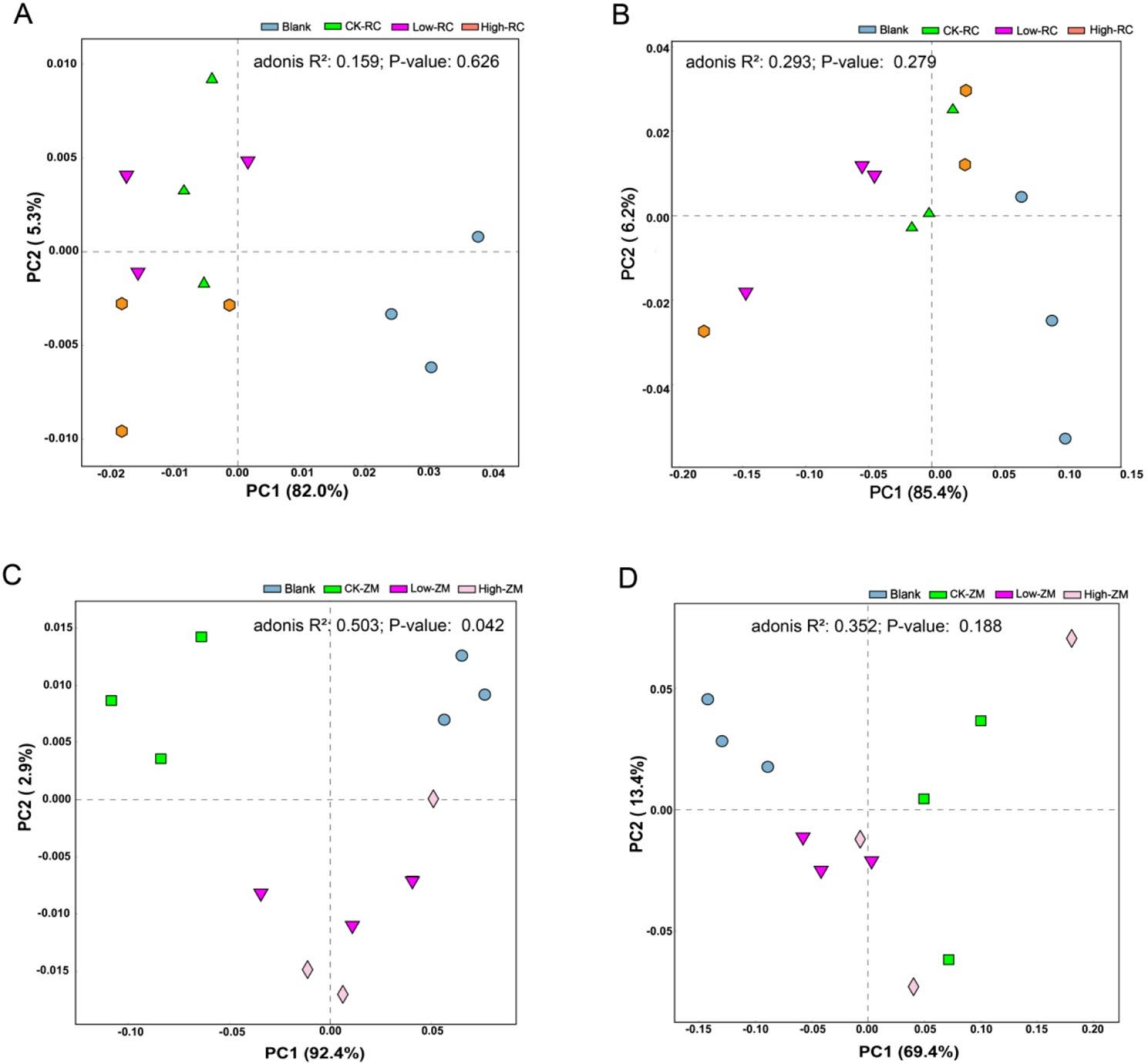
PCA of soil microbiota β-diversity between DBP-treated and initial soil. Note: (**A**) Principal components analysis based on Bray-Curtis dissimilarity of 16S rDNA gene amplicon sequences (V4-V5 region) for variety RC69. **B** Principal components analysis based on Bray-Curtis dissimilarity of fungal ITS gene amplicon sequences (ITS1 region) for variety RC69. **C** Principal components analysis based on Bray-Curtis dissimilarity of 16S rDNA gene amplicon sequences (V4-V5 region) for variety ZM7698. **D** Principal components analysis based on Bray-Curtis dissimilarity of fungal ITS gene amplicon sequences (ITS1 region) for variety ZM7698. Blank, not planting; CK-RC, RC69 rhizosphere soil without DBP treatment; Low-RC, RC69 rhizosphere with low concentration DBP treatment; High-RC, RC69 rhizosphere with high concentration DBP treatment; Low-ZM, ZM7698 rhizosphere with low concentration DBP treatment; High-ZM, ZM7698 rhizosphere with high concentration DBP treatment. Adonis results (R², p) shown in each panel are based on comparisons among CK, Low, and High only (Blank not included)

To explore the microbiological mechanism underlying the differential responses to DBP stress between the tolerant variety RC69 and the sensitive variety ZM7698, the rhizosphere microbiome under different DBP concentration treatments was analyzed by Illumina high-throughput sequencing in this study. Correlation network analysis revealed that the rhizosphere microbiota of wheat variety RC69 maintained a bacterial-dominant structure (the proportion of bacteria in the high-connectivity nodes was > 50%) after DBP treatments. In contrast, ZM7698 exhibited a significant DBP-induced community transition, with the proportion of fungal nodes among highly connected nodes increasing from 33.3% to 60% (Fig. 6). Principal component analysis (PCA) demonstrated that the β diversity of rhizosphere bacteria in variety RC69 was significantly affected by planting effects and almost unaffected by DBP treatment (Fig. 7A); The variety ZM7698 was significantly affected by both planting and DBP treatment (Fig. 7C); There was no significant separation of rhizosphere soil fungi in all treatments of the two varieties (Fig. 7B and D). In summary, the rhizosphere bacterial community of the tolerant variety RC69 exhibited greater stability, while DBP stress significantly changed the rhizosphere bacterial community structure of the sensitive variety ZM7698.

**Fig. 8:**
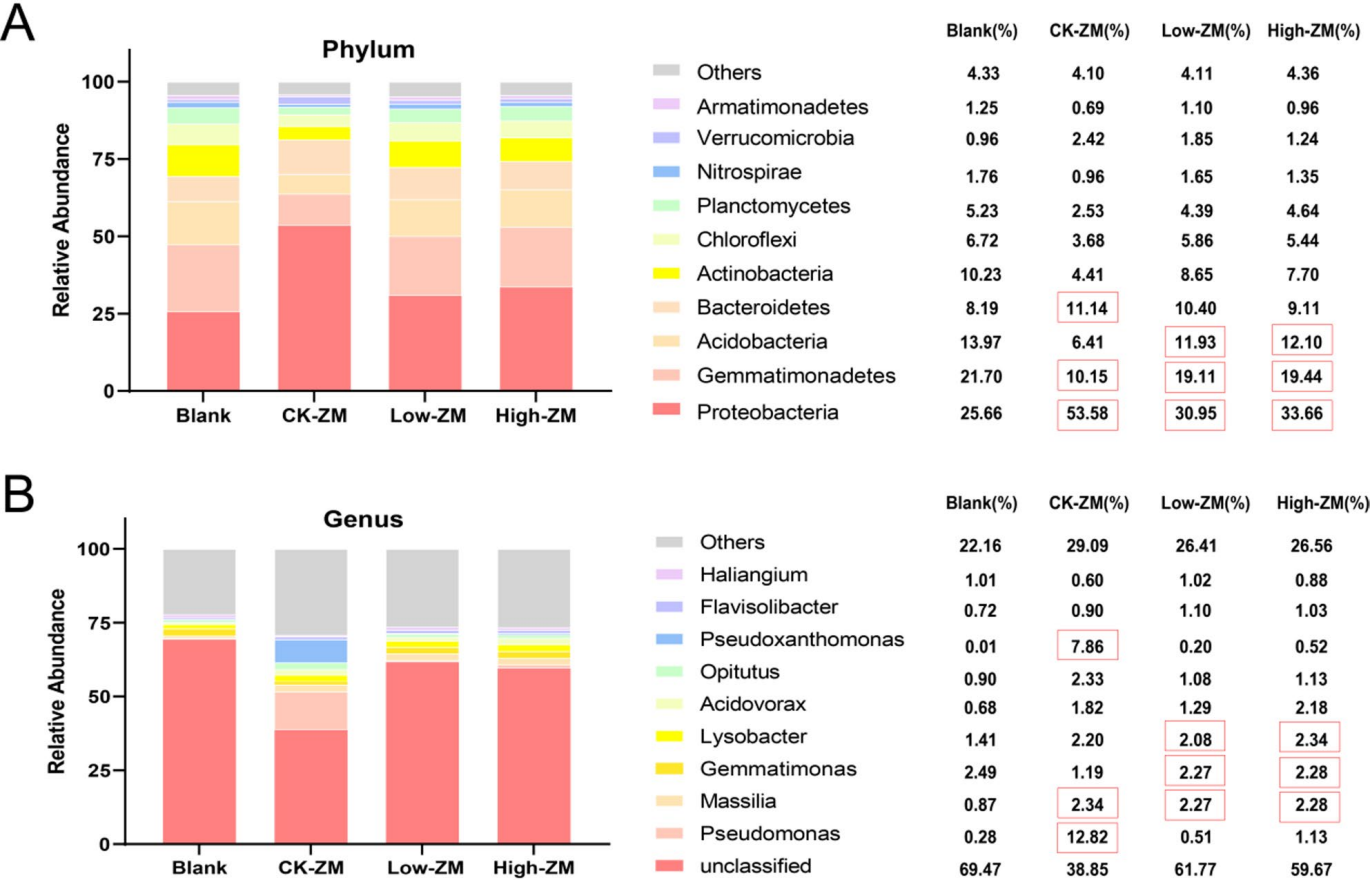
Abundance analysis of soil bacteria at phylum level and genus level. Note: (**A**) Abundance analysis of soil bacteria at phylum level; (**B**) Abundance analysis of soil bacteria at genus level. Stack plots were created with the top ten microbes in each sample in terms of abundance, with the rest categorized as others. Blank, not planting; CK-ZM, not treated with DBP; Low-ZM, low DBP treatment; High-ZM, high DBP treatment

On this basis, the bacterial community structure of rhizosphere soil of wheat variety ZM7698 under different treatments was further analyzed. At the phylum level, the top three dominant bacterial phyla in the control group were Proteobacteria, Gemmatimonadetes, and Bacteroidetes, while in the low DBP and high DBP treatment groups, Acidobacteria replaced Bacteroidetes to rank among the top three (Fig. 8A). At the genus level, the top three dominant bacterial genera in the control group were *Pseudomonas*, *Massilia*, and *Pseudoxanthomonas*, while the top three dominant bacteria in the DBP treatment groups were *Massilia*, *Gemmatimonas*, and *Lysobacter* (Fig. 8B). These results indicate that DBP stress was associated with significant changes in the abundance of dominant bacterial taxa in the rhizosphere of the sensitive wheat variety ZM7698.

### Differential microorganisms and differential biomarkers under DBP stress

**Fig. 9:**
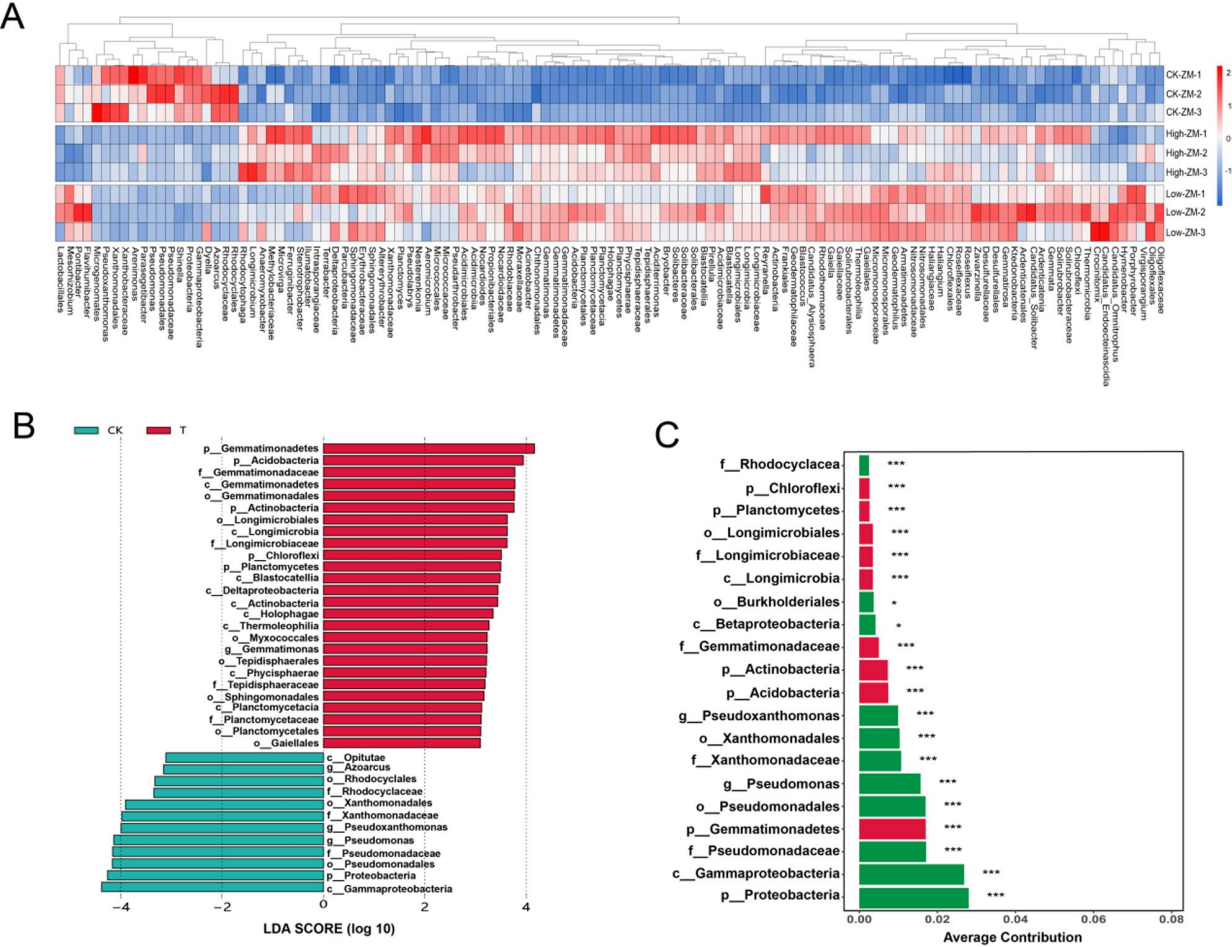
Screening and analysis of differential microorganisms and marker microorganisms. Note: (**A**) Heatmap showing the relative abundance of differential microorganisms in the plant rhizosphere under different DBP treatments. Rows represent treatment groups, and columns represent microbial genera. CK-ZM, not treated with DBP; Low-ZM, low DBP treatment; High-ZM, high DBP treatment. **B** Distribution of LDA scores for significantly differential biomarkers. Green bars represent taxa enriched in the control group, while red bars represent taxa enriched in the DBP-treated group. **C** The SIMPER analysis displays the top 20 species contributing the most to the differences between groups, along with their average contribution percentages. Green bars indicate taxa enriched in the control group, while red bars represent taxa enriched in the DBP-treated group. Species contribution rates were validated by 999 permutation tests, with significant contributors marked by “*” for *p* < 0.05 and “***” for *p* < 0.01.”

A total of 121 significantly differentiated microbial genera were identified by comparing the rhizosphere microbial communities of wheat variety ZM7698 under different DBP concentration treatments (Fig. 9A). Among these, 16 genera were predominantly enriched in the control group, 93 genera were enriched in the DBP treatment groups, and 8 were exclusively enriched in the low DBP treatment group (Fig. 9A). These results demonstrate that the abundance of rhizosphere soil microorganisms in ZM7698 varies significantly under different DBP concentrations.

The Linear Discriminant Analysis Effect Size (LEfSe) identified characteristic biomarkers for different treatment groups. Gammaproteobacteria, Proteobacteria, Pseudomonadales, and Pseudomonadaceae served as characteristic biomarkers for the control group (LDA score > 4), while Gemmatimonadetes was identified as a characteristic biomarker for the DBP-treated groups (Fig. 9B). The SIMPER analysis quantified the major taxa that drove the microbial community differences between the control group and treatment group, revealing that the Proteobacteria (average contribution 2.80%) and Gammaproteobacteria (average contribution 2.68%) were the key phylum and class that distinguished the DBP-contaminated group from the control group (Fig. 9C). At the genus level, *Pseudomonas* (average contribution 1.57%) and *Pseudoxanthomonas* (average contribution 0.99%) were the key genera that distinguished DBP-contaminated group from control group (Fig. 9C).

### Response of rhizosphere soil bacterial community function to DBP stress in wheat variety ZM7698

**Fig. 10:**
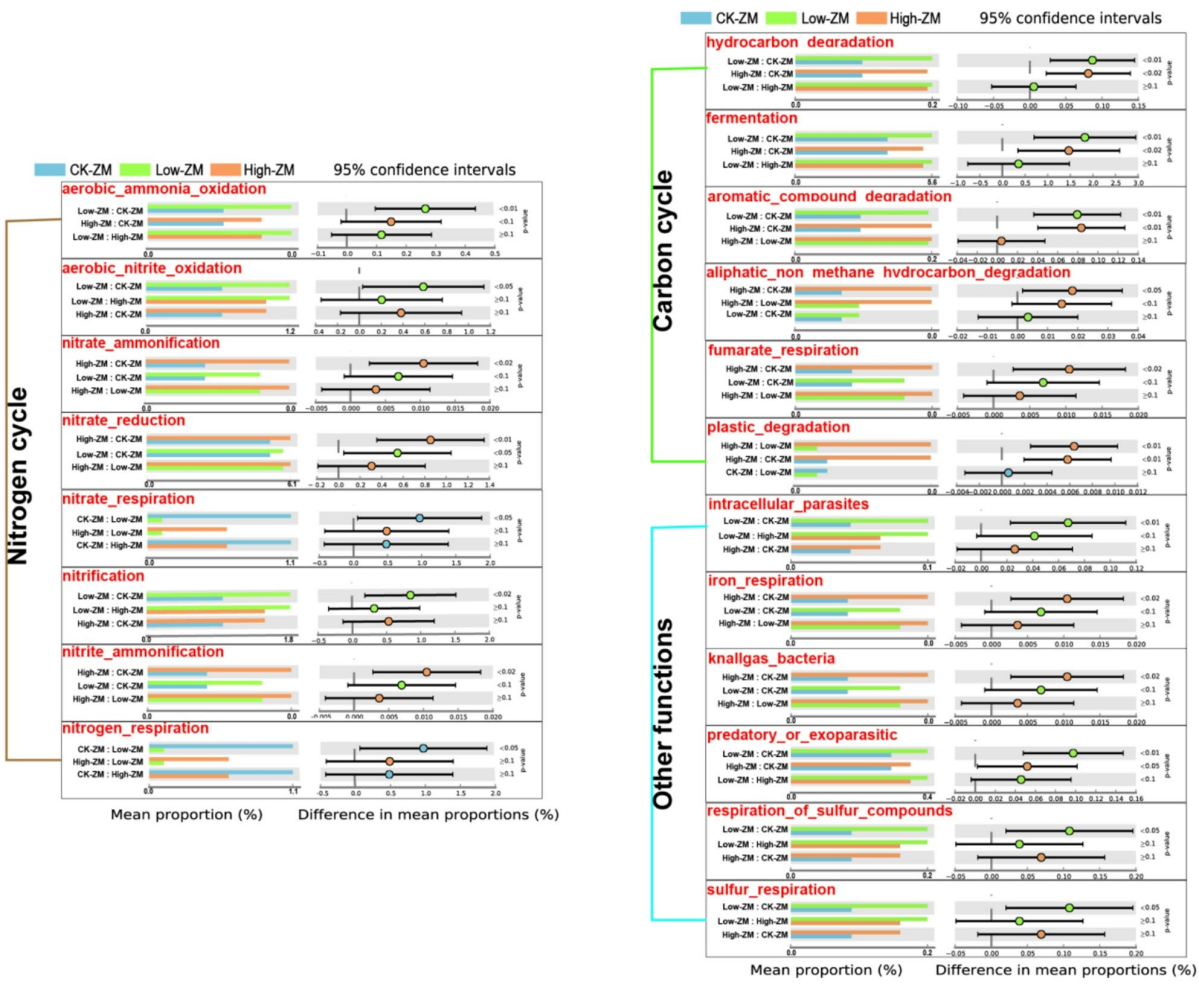
Functional analysis of prokaryotic microorganisms under DBP stress. Note: The image shows the functional differences in the rhizosphere bacterial community of wheat variety ZM7698 under DBP stress. CK-ZM, not treated with DBP; Low-ZM, low DBP treatment; High-ZM, high DBP treatment

Functional prediction analysis based on FAPROTAX was performed to infer the potential ecological functions of rhizosphere bacterial communities in the DBP-sensitive wheat variety ZM7698. Results showed that DBP stress significantly altered the predicted functional profiles of the rhizosphere bacterial community. A total of 20 functional taxa showed significant differences between the control and DBP-treated groups (Welch’s t-test, *P* < 0.05), including 8 nitrogen cycle-related functions, 6 carbon cycle-related functions, and 6 other functions (Fig. 10). Notably, the relative abundance of plastic-degrading bacteria significantly increased in the high DBP treatment group. In addition to plastic degradation, several other functional bacterial groups exhibited significantly higher abundance under high DBP stress, including aliphatic non methane hydrocarbon degradation, fumarate respiration, iron respiration, knallgas bacteria, nitrite ammonification, nitrate reduction, and nitrate ammonification (Fig. 10). These results indicate that DBP stress not only affects the composition of the rhizosphere microbiome, but also influences the ecological potential of the rhizosphere soil.

## Discussion

In this study, six priority-controlled PAEs were analyzed in 156 farmland soils in central Henan Province, and the results showed that DBP and DEHP were the main contaminants, with DBP exhibiting particularly significant contributions. This finding is consistent with reports from multiple agricultural regions worldwide [38–40]. From a physicochemical perspective, DEHP and DBP possess relatively high molecular weights, low water solubility, and low volatility, making them difficult to biodegradation in soil [41, 42]. These properties may partly explain their high residual levels in farmland soils of Henan Province.

Analysis of PAEs contamination in wheat grains revealed that PAEs were detected in all samples, suggesting that PAEs can enter the food chain through the soil-crop system. Furthermore, consistent with the distribution characteristics of PAEs in soils, DBP and DEHP were also identified as the predominant contaminants in wheat grains, indicating that these two compounds were not only widely present in soil, but also had strong bioaccumulation capacity. This finding is consistent with the observations reported by Sun et al. (2021) in Shanxi Province, which showed that the total PAEs (∑PAEs) concentration in wheat grains ranged from 445 to 764 µg/kg, and DEHP and DBP were the main pollutants [43]. Notably, the average concentration of DBP detected in wheat grains in this study was 0.510 mg/kg, which has exceeded the maximum residue limit for DBP in food issued by the General Office of the Ministry of Health of China in 2011 (Letter No. 551 [2011]) [44]. Although this wheat grain still needs a series of processing before it can be consumed, the potential health risks warrant serious attention given wheat’s status as a staple food with long-term dietary exposure implications.

This study evaluated the tolerance of 51 wheat varieties to DBP stress and found that DBP significantly inhibited wheat germination, root elongation, and seedling growth in a genotype-dependent manner. These results are consistent with previous studies on the phytotoxicity of DBP to wheat [8, 32] and further elucidate, at the varietal level, the diversity of crop responses to such pollution stress. The growth inhibition of plants by DBP may be related to its disruption of endogenous hormone balance and energy metabolism processes [45, 46]. Additionally, this study found that under DBP stress, the tolerant variety RC69 accumulated less DBP in its roots compared to the sensitive variety ZM7698, but exhibited significantly higher DBP content in its leaves than ZM7698. This phenomenon suggests a possible tolerance mechanism in RC69 involving rhizosphere-assisted reduction of DBP uptake and rapid transport of DBP to leaf tissues for vacuolar sequestration, thereby alleviating root toxicity. However, this hypothesis requires further direct testing using isotope tracing or subcellular localization studies. In contrast, the sensitivity of ZM7698 may be due to insufficient transport and compartmentalization capability, resulting in continuous accumulation of DBP in its roots, which directly damages cell structure and function, ultimately leading to systemic growth inhibition.

Given the role of rhizosphere microbial communities in plant stress resistance [47], we investigated whether the differential tolerance of wheat varieties to DBP is associated with shifts in their rhizosphere microbiota. Co-occurrence network analysis revealed that under DBP stress, the tolerant variety RC69 maintained a bacterial-dominated and structurally stable rhizosphere microbial community. In contrast, the sensitive variety ZM7698 underwent a structural shift in its network, marked by a decline in bacterial dominance and a rise in the importance of fungal nodes. Previous studies have shown that bacteria-dominated rhizosphere microbial communities are often associated with healthy plants, whereas an increased fungal proportion may indicate stress response or disease occurrence [48–50]. Therefore, the maintenance of a bacteria-dominated rhizosphere microbial environment in RC69 may be associated with its DBP tolerance. PCA analysis further confirmed the stability of the rhizosphere microbial community in the tolerant variety RC69. In contrast, the rhizosphere bacterial community of ZM7698 was significantly affected by DBP treatment. It is worth noting that the fungal communities of both varieties did not exhibit significant DBP-responsive segregation. One possible explanation is that, compared with bacterial cell walls, fungal cell walls possess a more complex and denser structure (e.g., rich in chitin and β‑glucans), which may act as an effective barrier against hydrophobic DBP molecules and reduce their toxicity toward fungal communities. In addition, fungi may exhibit a lagged response to DBP due to their relatively longer life cycles and slower community turnover rates compared with bacteria.

In-depth analysis of the sensitive variety ZM7698 revealed distinct successional changes in its rhizosphere bacterial community under DBP stress. At the phylum level, the relative abundance of Acidobacteria increased, displacing Bacteroidetes as the dominant group—a shift potentially linked to DBP-induced soil acidification [51], given the well-documented acid-tolerant nature of Acidobacteria [52]. At the genus level, DBP treatment resulted in a substantial decrease in the relative abundance of *Pseudomonas* and *Pseudoxanthomonas*, with reductions of 91.1% and 87.7%, respectively (Fig. 8B). The former has been reported to disappear from the rhizosphere under DBP stress [53], while the decline of the latter may be attributed to the inhibition of its metabolic activity by DBP. Although *Pseudoxanthomonas* has been shown to degrade DEHP [54], its ability to degrade DBP requires further investigation. The LEfSe analysis further revealed that Gammaproteobacteria, Proteobacteria, Pseudomonadales, and Pseudomonadaceae were characteristic biomarkers of DBP-untreated soil, while Gemmatimonadetes was significantly enriched in the DBP-treated group (Fig. 9B). These differences suggest that DBP contamination may lead to the succession of soil microbial communities to specific taxa, and some bacteria within Gemmatimonadetes may possess the ability to degrade DBP. The Simper analysis further quantified the key driver taxa and identified Proteobacteria, Gammaproteobacteria, and Pseudomonas as the primary phylum, class, and genus distinguishing the DBP-contaminated group from the control group, indicating that they may play an important role in DBP stress response.

DBP contamination not only altered the species abundance of the soil microbial community but also affected its ecological functions in the rhizosphere. Previous studies have shown that DBP pollution increases the gene abundance related to carbon, nitrogen, and sulfur metabolism in the short term [55], which is similar to the results of this study. Functional profiles predicted in this study suggested that DBP stress was associated with significant shifts in the metabolic functions of microbial communities, particularly those related to nitrogen and carbon cycling (Fig. 10). The marked increase in plastic-degrading bacteria may result from DBP’s structural properties, with higher concentrations selecting for microbes with degradation potential. Additionally, the abundance of functional bacteria involved in aliphatic non-methane hydrocarbon degradation, fumarate respiration, and iron respiration also increased notably, indicating that DBP stress may promote the metabolic activity of certain anaerobic or facultative anaerobic microorganisms. The increased abundance of functional bacteria involved in nitrite ammonification, nitrate reduction, and nitrate ammonification may lead to the accumulation of ammonium nitrogen in the soil. Due to the uptake preference of wheat for nitrate nitrogen [56], this nitrogen form change may indirectly interfere with crop nutrient absorption by affecting nitrogen availability. It should be noted that these functional predictions were performed using FAPROTAX, which infers microbial functions based on annotated cultured strains and thus may underestimate the functional potential of uncultured taxa. In future studies, metagenomics or metatranscriptomics could be used to verify these predicted changes.

Several limitations of this study should be acknowledged. First, the greenhouse experiments were conducted under controlled conditions, and field trials are needed to confirm the applicability of our findings under more complex environmental conditions. Second, the tolerance assessment was primarily based on biomass accumulation at the seedling stage (day 30), which may not fully recapitulate plant performance throughout the entire life cycle. Third, the measured DBP concentrations were considerably lower than the nominal spiking levels, reflecting the rapid dissipation of DBP during soil equilibration, which should be considered when interpreting the exposure-response relationships. Additionally, this study focused on bacterial communities and their potential functions, potentially overlooking the roles of fungal communities in DBP tolerance.

## Conclusion

This study demonstrates that wheat varieties exhibit significant differences in their tolerance to DBP stress, and that these differences are closely related to their genetic backgrounds and the stability of their rhizosphere microbial communities. Under DBP stress, the tolerant variety RC69 maintained stable rhizosphere microbial community structure and related ecological functions, whereas the sensitive variety ZM7698 exhibited significant disruption. Moreover, the enrichment of potential degrading bacteria in the sensitive variety suggests a promising avenue for microbial remediation. This study also identified key differential microbial taxa associated with DBP tolerance, laying a foundation for subsequent functional validation and targeted regulation. Future research should focus on elucidating the ecological functions of these taxa and exploring synthetic biology strategies to engineer rhizosphere microbial communities, thereby improving crop resistance to DBP contamination.

## Data Availability

Soil microbiome data that support the findings of this study are available in the SRA at the National Center for Biotechnology Information (NCBI) with the identifier PRJNA1301106 and PRJNA1301118.
